# DNA Methylation of *IGF2DMR* and *H19* Is Associated with Fetal and Infant Growth: The Generation R Study

**DOI:** 10.1371/journal.pone.0081731

**Published:** 2013-12-12

**Authors:** Marieke I. Bouwland-Both, Nina H. van Mil, Lisette Stolk, Paul H. C. Eilers, Michael M. P. J. Verbiest, Bastiaan T. Heijmans, Henning Tiemeier, Albert Hofman, Eric A. P. Steegers, Vincent W. V. Jaddoe, Régine P. M. Steegers-Theunissen

**Affiliations:** 1 The Generation R Study Group, Erasmus Medical Centre, Rotterdam, The Netherlands; 2 Department of Obstetrics and Gynecology, Erasmus Medical Centre, Rotterdam, The Netherlands; 3 Department of Child and Adolescent Psychiatry/Psychology, Erasmus Medical Centre, Rotterdam, The Netherlands; 4 Department of Internal Medicine, Erasmus Medical Centre, Rotterdam, The Netherlands; 5 Netherlands Consortium of Healthy Aging, Rotterdam/Leiden, The Netherlands; 6 Department of Biostatistics, Erasmus Medical Centre, Rotterdam, The Netherlands; 7 Department of Molecular Epidemiology, Leiden University Medical Centre, Leiden, The Netherlands; 8 Department of Epidemiology, Erasmus Medical Centre, Rotterdam, The Netherlands; 9 Department of Pediatrics, Erasmus Medical Centre, Rotterdam, The Netherlands; 10 Department of Clinical Genetics, Erasmus Medical Centre, Rotterdam, The Netherlands; CNRS, France

## Abstract

Changes in epigenetic programming of embryonic growth genes during pregnancy seem to affect fetal growth. Therefore, in a population-based prospective birth cohort in the Netherlands, we examined associations between fetal and infant growth and DNA methylation of *IGF2DMR*, *H19* and *MTHFR*. For this study, we selected 69 case children born small-for-gestational age (SGA, birth weight <-2SDS) and 471 control children. Fetal growth was assessed with serial ultrasound measurements. Information on birth outcomes was retrieved from medical records. Infant weight was assessed at three and six months. Methylation was assessed in DNA extracted from umbilical cord white blood cells. Analyses were performed using linear mixed models with DNA methylation as dependent variable. The DNA methylation levels of *IGF2DMR* and *H19* in the control group were, median (90% range), 53.6% (44.5–61.6) and 30.0% (25.6–34.2) and in the SGA group 52.0% (43.9–60.9) and 30.5% (23.9–32.9), respectively. The MTHFR region was found to be hypomethylated with limited variability in the control and SGA group, 2.5% (1.4–4.0) and 2.4% (1.5–3.8), respectively. SGA was associated with lower *IGF2DMR* DNA methylation (β = −1.07, 95% CI −1.93; −0.21, P-value = 0.015), but not with *H19* methylation. A weight gain in the first three months after birth was associated with lower *IGF2DMR* DNA methylation (β = −0.53, 95% CI −0.91; −0.16, P-value = 0.005). Genetic variants in the *IGF2/H19* locus were associated with *IGF2DMR* DNA methylation (P-value<0.05), but not with *H19* methylation. Furthermore, our results suggest a possibility of mediation of DNA methylation in the association between the genetic variants and SGA. To conclude, *IGF2DMR* and *H19* DNA methylation is associated with fetal and infant growth.

## Introduction

One of the main causes of perinatal morbidity and mortality is being born small-for-gestational age (SGA) [1], [2]. SGA can be the result of a poor prenatal environment, which could have been induced by an adaptive response during fetal life [3]. These infants are particular at risk for early onset of non-communicable diseases when they also experience a period of catch up growth during the first months of life [4]. One of the proposed underlying mechanisms are changes in epigenetic markings acquired and maintained during pregnancy, which are not directly related to the DNA sequence itself [5].

One of the best understood epigenetic mechanisms are modifications in DNA methylation which occur predominantly at cytosines of CpG dinucleotides [5]. To establish and maintain DNA methylation patterns, methyl donors, such as folate and choline, acting as intermediates in the one-carbon pathway are required [6]. Low intake of methyl donors and subtle genetic variations in methyltetrahydrofolate reductase (MTHFR) can derange the one carbon pathway resulting in a mild to moderate hyperhomocysteinemia. Associations have been reported between elevated maternal homocysteine levels during pregnancy and a lower birth weight and increased risk of SGA [7]. Moreover, hyperhomocysteinemia also affects DNA methylation levels [8], [9].

Although DNA methylation patterns are believed to be relatively stable, it seems that exposures, particularly during the prenatal period, can permanently alter DNA methylation patterns in offspring [10]. Especially genes that are expressed in a parent-of-origin-specific manner, known as imprinted genes, are of interest as they are essential during development and regulated through epigenetic mechanisms [11]. The *IGF2/H19* imprinted region is one of the best studied loci. In humans, exposure to famine during the periconceptional period has been linked to altered DNA methylation patterns of the insulin-like growth factor 2 (*IGF2DMR*) in adulthood [12]. Also, periconceptional folic acid supplement use is associated with increased methylation of the *IGF2DMR* in humans and *IGF2DMR* methylation was inversely associated with birth weight [13].

We hypothesized that changes in fetal growth and subsequent early infant growth are partly due to alterations in DNA methylation of genes implicated in fetal and infant growth. Therefore, we assessed DNA methylation in 2 imprinted genes (*IGF2* and *H19*) and 1 non imprinted folate gene (*MTHFR*). Our main focus was to investigate the association between SGA and DNA methylation. Next, we examined associations between fetal and infant growth and DNA methylation. In addition, we investigated whether genetic variations within the investigated genes are associated with DNA methylation. Furthermore, we explored the possible mediation of DNA methylation in the association between the genetic variants and SGA.

## Materials and Methods

### Design and study population

This study was embedded in the Generation R study, a population-based cohort study from early fetal life onwards in Rotterdam, the Netherlands [14]. The study has been approved by the Medical Ethical Committee of the Erasmus Medical Center in Rotterdam (MEC 198.782/2001/31). Written consent was obtained from all participating mothers for both maternal and child data. Mothers were enrolled during pregnancy, between 2001 and 2005. In total, 8880 mothers were enrolled during pregnancy.

The present study was carried out in a subset of the original Generation R cohort (n = 540). Analyses were restricted to Dutch singleton pregnancies (n = 4,829) and infants with available DNA extracted from umbilical cord white blood cells (n = 3,127). From this study group all infants born with a gestational-age and sex-adjusted birth weight below −2 standard deviation score (SDS) (n = 69) were selected for analysis. The current study is part of a project, in which we investigate the hypothesis that both SGA and children with attention deficit hyperactivity disorder (ADHD) have a shared causality in DNA methylation of especially imprinted fetal growth genes. Therefore, 92 children were included with ADHD based on parent interview Diagnostic Inventory of Screening Children or Child Behavior Checklist teacher report at age 6. Two of the infants with ADHD were also born with a gestational-age and sex-adjusted birth weight below −2 SDS. The remaining 381 control infants were randomly selected. Therefore, the control group consisted of 90 ADHD children and 381 infants who were randomly drawn.

### Fetal and infant growth

Fetal ultrasound measurements were performed to assess gestational age and to estimate fetal growth [15]. Crown-to-rump length was used for pregnancy dating until a gestational age of 12 weeks and 5 days (crown-to-rump length<65 mm), and biparietal diameter (BPD) for pregnancy dating thereafter (gestational age from 12 weeks and 5 days onwards, BPD> 23 mm). Fetal growth characteristics included head circumference, BPD, abdominal circumference, and femur length and were measured trans-abdominal to the nearest millimeter using standardized ultrasound procedures in mid pregnancy (median 20.5 weeks of gestation, 90% range 19.1–22.4) and late pregnancy (median 30.4 weeks of gestation, 90% range 28.8–32.1). Estimated fetal weight (EFW) was calculated using the formula by Hadlock [16]. A gestational age-adjusted SDS, based on reference growth curves from the entire study population, was constructed for EFW [15].

Information concerning date of birth, infant sex and birth weight was obtained from the medical records of community midwives and hospitals [14]. Gestational age and sex-adjusted SDS were constructed for birth weight, according to the methodology of Niklasson et al [17]. SGA was defined according to the International SGA Advisory Board Panel as a gestational age and sex-adjusted birth weight below −2 SDS [18]. Born appropriate for gestational age (AGA) was defined as a gestational age and sex-adjusted birth weight ≥−2 SDS. Born large for gestational age (LGA) was defined as a gestational age and sex-adjusted birth weight ≥2 SDS. The growth rate of weight between mid-pregnancy and birth was calculated as (birth weight SDS - EFW SDS in mid pregnancy).

Trained staff in community health centers obtained information on infant weight during periodic visits scheduled at three (median 3.3 months, 90% range 3.0–3.9) and six months (median 6.2 months, 90% range 5.5–7.4) [14]. For every visit, an age- and sex-adjusted SDS for weight was calculated with the use of Dutch reference curves (Growth Analyzer 3.0, Dutch Growth Research Foundation, Rotterdam, the Netherlands). Infant growth rates of weight gain in the first three and six months after birth were calculated as (weight at three or six months SDS – birth weight SDS).

### Assessment of DNA methylation and genotyping

Genomic DNA was isolated from umbilical cord white blood samples at birth as previously been described [19]. Based on previous studies, three loci were selected for the assessment of DNA methylation [9], [20]. DNA methylation of *IGF2DMR* and *H19* have been previously studied by other groups [12], [20]. However, DNA methylation *MTHFR* has not been studied before. It was chosen as it is a key enzyme in the one carbon pathway. A CpG island outside the *MTHFR* gene was selected for analyses. Primers were designed using the online tool of MySequenom.com. Details of the measured amplicons can be found in Table S1. Isolated genomic DNA (500 ng) was treated with sodium bisulphite for 16 hours using the EZ-96 DNA methylation kit (Shallow) (Zymo Research, Irvine, CA, USA), according to the manufactures' protocol. Samples were randomly distributed on six 96-well plates. The bisulphite treatment was followed by PCR amplification, fragmentation after reverse transcription and analysis on a mass spectrometer, according to the manufactures' protocol (Sequenom, Inc, San Diego, USA). This generated mass signal patterns that were translated into quantitative DNA methylation levels of different CpG sites of the selected genes by MassARRAY EpiTYPER Analyzer software (v1.0, built 1.0.6.88 Sequenom, Inc, San Diego, USA) [21], [22]. Fragments containing one or more CpG sites were called CpG units. PCR and subsequent steps were done in triplicate.

Data quality control for methylation consisted of exclusion of CpG units with too low or too high mass or CpG units with overlapping or duplicate RNA fragments (e.g. silent signals) were excluded from further analysis. Furthermore, at least two out of three of the replicate measurements per CpG unit had to be successful, the standard deviation of the duplicates or triplicates had to be ≤0.10 and the success rate per CpG unit had to be ≥75%. Last, CpG units that contained a known SNPs with a frequency >5% were also excluded, as this could change the weight of the CpG unit and therefore interfere with the measurement. Details concerning the success rate of the amplicons can be found in Table S2.

Based on literature [23], four SNPs in the *IGF2/H19* locus were identified that were associated with birth weight in Caucasian children. Subsequently, two genetic variants of the *MTHFR* gene which have been associated with global DNA methylation were identified [24]. Genotypes were obtained using high-density SNP arrays (Illumina) and then imputed for ∼2.4 million HapMap SNPs (Phase II, release 21/22). Frequency distribution conformed to the Hardy-Weinberg equilibrium. Details concerning the SNPs can be found in Table S3.

### Covariates

From self-administered questionnaires, data was available on maternal age and maternal educational level, parity, smoking and folic acid supplement use before and during pregnancy. Maternal education level was assessed by the highest completed education and classified as 1) none/primary or ‘low’; 2) secondary or ‘medium’; 3) college/university or ‘high’. Parity was classified as (1) nulliparous and (2) multiparous. Maternal smoking was assessed in each trimester. Women, who reported any or no smoking during pregnancy were respectively classified as ‘smokers’ and ‘non-smokers’. Folic acid supplement use was categorized into 1) folic acid supplement use (pre- or postconceptional start); 2) no folic acid supplement use. At enrolment (median 13.5 weeks, 90% range 10.7; 21.6) maternal weight and height were measured to calculate body mass index (BMI, kg/m^2^). From hospital medical records, the occurrence of hypertension and hypertension-related pregnancy disorders were obtained [25]. Preeclampsia was defined according to the criteria described by the International Society for the Study of Hypertension in Pregnancy (ISSHP) [26].

### Statistical analysis

First, we tested for differences in maternal or infant characteristics between cases and controls using chi-square, Mann-Whitney U and T-tests. Second, linear mixed models were used to examine the associations between fetal or infant growth (independent variable) and DNA methylation (dependent variable). This model was chosen as it can account for correlation between CpG dinucleotides, incorporates relevant adjustments within the models and has the ability to accommodate missing data. The restricted maximum likelihood (REML) method was used for the model fitting. DNA methylation was treated as a continuous variable. To achieve normality, DNA methylation was square root transformed. Outliers per CpG (>3SD) were excluded from further analysis. For all analyses, subject/person identifier was added as random effect and bisulphite batch and CpG site were added as fixed effects. In the crude analyses, the growth characteristic was entered as a fixed effect. Potential confounders were additionally entered to the model at the same time as fixed effects. In addition, the analyses were also repeated with exclusion of the ADHD and the LGA cases. Third, associations between genetic variants in the *IGF2/H19* locus and the *MTHFR* gene and DNA methylation levels (dependent variable) were investigated using a linear mixed model. The genotype was entered to the model continuously as a fixed effect. Subsequently, we explored the possibility of mediation of DNA methylation in the association between the genetic variant and SGA using logistic regression models.

Missing data of maternal educational level (1.3%), folic acid supplement use (18.5%) and smoking (6.3%) were completed using the Markov-Chain-Monte-Carlo multiple imputation technique [27]. Ten imputed datasets were created. For all analyses, results including imputed missing data are presented. Multiple testing correction was performed according to the method developed by Bonferroni. The linear mixed model analyses were performed using the data measured in triplicates. All analyses were performed using the Statistical Package of Social Sciences version 20.0 for Windows (SPSS Inc, Chicago, IL, USA).

## Results

The maternal and fetal characteristics are presented in table 1. Mothers of children born SGA were lower educated, more often nulliparous, smoked more often during pregnancy, used less often a folic acid supplement during the periconceptional period and more frequently developed preeclampsia (all P-value<0.05). The median (90% range) DNA methylation levels of *IGF2DMR*, *H19* and *MTHFR* were 53.2% (44.3–61.3), 30.1% (25.5–34.2) and 2.5% (1.4–4.0) respectively. The median (90% range) DNA methylation levels of *IGF2DMR*, *H19* and *MTHFR* in the AGA group were 53.6 (44.5–61.6), 30.0 (25.6–34.2) and 2.5 (1.4–4.0), respectively. The median (90% range) DNA methylation levels of *IGF2DMR*, *H19* and *MTHFR* in the SGA group were 52.0 (43.9–60.9), 30.5 (23.9–32.9) and 2.4 (1.5–3.8), respectively. As the MTHFR region was found to be hypomethylated with limited variability, no further analyses were conducted within this region.

**Table 1:** Maternal and infant characteristics.

	All infants n = 540	Controls n = 471	SGA n = 69	P-value^1^
**Maternal characteristics**				
Age at intake (years)*	30.3 (5.1)	30.3 (5.1)	30.0 (5.4)	NS
Body mass index at intake (kg/m^2^)†	23.3 (19.3–32.4)	23.3 (19.4–32.4)	22.8 (18.9–30.9)	NS
Education (%)				*0.003*
*Primary education*	4.4	3.8	8.7	
*Secondary education*	45.9	45.9	46.4	
*Higher education*	48.3	49.3	42.0	
*Missing*	1.3	1.1	2.9	
Parity (%)				*<0.001*
*0*	66.9	64.1	85.5	
≥*1*	33.1	35.9	14.5	
Smoking status during pregnancy (%)				*0.003*
*Yes*	23.1	21.9	31.9	
*Until pregnancy recognition*	8.7	8.5	10.1	
*No*	61.9	63.3	52.2	
*Missing*	6.3	6.4	5.8	
Folic acid supplement use during pregnancy (%)				*<0.001*
*Start preconception*	43.5	45.2	31.9	
*Start postconception*	25.9	24.8	33.3	
*No*	12.0	11.5	15.9	
*Missing*	18.5	18.5	18.8	
Preeclampsia (%)	2.0	1.3	7.2	*<0.001*
**Infant characteristics**				
Gender (% boys)	57.8	57.3	60.9	NS
Estimated fetal weight (grams), mid-pregnancy†	358.2 (257.7–537.4)	359.5 (364.3–544.7)	352.5 (238.1–502.4)	*<0.001*
Estimated fetal weight (grams), late-pregnancy†	1538.2 (1199.1–2032.6)	1589.4 (1251.5–2038.2)	1362.9 (1118.6–1564.3)	*<0.001*
Gestational age at birth (weeks)†	40.3 (37.6–42.1)	40.3 (37.4–42.1)	40.3 (37.7–42.1)	NS
Birth weight (grams)†	3418 (2491–4300)	3510 (2735–4325)	2625 (1930–2830)	*<0.001*
Weight (grams), 3 months†	6215 (4990–7541)	6260 (5120–7613)	5690 (4823–6545)	*<0.001*
Weight (grams), 6 months†	7690 (6320–9255)	7820 (6450–9330)	7085 (5890–9250)	*<0.001*
*IGF2DMR* methylation (%)†	53.2 (44.3–61.3)	53.6 (44.5–61.6)	52.0 (43.9–60.9)	*0.003*
*H19* methylation (%)†	30.1 (25.5–34.2)	30.0 (25.6–34.2)	30.5 (23.9–32.9)	NS

Values are presented as

* mean (standard deviation) and

†median (90% range).

1 Student's T-test, Mann Whitney U and chi-square tests are used to test differences between the control group and the SGA group.

Table 2 shows the associations between the occurrence of SGA (categorical) and DNA methylation levels of *IGF2DMR* and *H19*. SGA was associated with lower *IGF2DMR* DNA methylation (β = −1.07, 95% CI −1.93; −0.21, P-value = 0.015). Expressed relative to the standard deviation, this difference corresponds with a standardized effect size in DNA methylation of −0.13 SDS units. No associations were observed between SGA and *H19* methylation. After multiple testing adjustment (three independent loci), the association of SGA with *IGF2DMR* methylation remained significant.

**Table 2:** Association between SGA and DNA methylation.

	*IGF2DMR* methylation (n = 499)			*H19* methylation (n = 510)		
	bèta^1^	95% CI	P-value	bèta^1^	95% CI	P-value
MODEL 1: adjusted for correlations between CpG sites, bisulphite batch, gestational age						
Small-for-gestational age (<−2 SDS)	**−1.26**	**−2.10; -0.42**	**0.003**	−0.15	−0.78; 0.48	0.635
MODEL 2: model 1 + maternal age, maternal educational level, parity and fetal gender						
Small-for-gestational age (<−2 SDS)	**−1.22**	**−2.07; −0.37**	**0.005**	−0.25	−0.90; 0.40	0.443
MODEL 3: model 2 + maternal BMI, folic acid supplement use, smoking and the occurrence of preeclampsia						
Small-for-gestational age (<−2 SDS)	**−1.07**	**−1.93; -0.21**	**0.015**	−0.27	−0.94; 0.39	0.422

Results from linear mixed model analyses with small-for-gestational age as independent variable and DNA methylation as dependent variable.

1 Analyses were performed with square-root transformed methylation data and values are presented as regression coefficients (95% confidence interval).

Table 3 shows the associations between fetal and infant growth and DNA methylation. As the SGA cases were oversampled in the data, the analyses were performed in the total study population, but were also stratified for SGA or AGA. In the total study population, lower *IGF2DMR* DNA methylation was associated with an increase in weight in the first three months after birth (β = −0.46, 95% CI −0.86; −0.07, P-value = 0.022), corresponding to a standardized difference in DNA methylation of −0.06 SDS. In the total study population, no association was found with *H19* methylation. After the analyses were stratified for SGA and AGA, higher *H19* methylation was associated with an increase in weight in the first three months after birth (β = 0.35, 95% CI 0.03; 0.68, P-value = 0.034) in children born AGA, which corresponds with a standardized difference in DNA methylation of −0.05 SDS.

**Table 3:** Associations between fetal and infant growth parameters and DNA methylation.

	Crude^1^	P-value	Adjusted^2^	P-value	Crude^1^	P-value	Adjusted^2^	P-value
ALL INFANTS	***IGF2DMR*** ** methylation (n = 499)**				***H19*** ** methylation (n = 510)**			
Birth weight SDS	0.15 (−0.08; 0.38)	0.206	0.05 (−0.21; 0.31)	0.704	−0.01 (−0.19; 0.16)	0.888	0.06 (−0.15; 0.26)	0.597
Δ weight: 2nd trimester – birth	−0.12 (−0.60; 0.37)	0.642	−0.13 (−0.64; 0.38)	0.606	−0.27 (−0.61; 0.07)	0.116	−0.19 (−0.54; 0.16)	0.283
Δ weight: birth-3 months	**−0.53 (−0.91; −0.16)**	**0.005**	**−0.46 (−0.86; −0.07)**	**0.022**	0.23 (−0.04; 0.50)	0.099	0.22 (−0.08; 0.52)	0.153
Δ weight: birth-6 months	−0.23 (−0.53; 0.08)	0.142	−0.16 (−0.50; 0.18)	0.367	0.16 (−0.06; 0.38)	0.146	0.11 (−0.15; 0.37)	0.407
SGA INFANTS	***IGF2DMR*** ** methylation (n = 65)**				***H19*** ** methylation (n = 65)**			
Birth weight SDS	1.55 (−0.31; 3.41)	0.103	1.18 (−0.83; 3.18)	0.251	−0.03 (−1.39; 1.33)	0.965	−0.08 (−1.80; 1.63)	0.924
Δ weight: 2nd trimester – birth	0.89 (−1.03; 2.80)	0.363	−0.02 (−3.68; 3.64)	0.992	**1.71 (0.55; 2.87)**	**0.004**	−1.09 (−9.41; 7.22)	0.790
Δ weight: birth-3 months	−1.26 (−2.92; 0.40)	0.136	−1.65 (−3.76; 0.47)	0.127	0.04 (−1.23; 1.31)	0.950	0.18 (−2.13; 2.48)	0.884
Δ weight: birth-6 months	−0.18 (−1.12; 0.76)	0.704	−0.07 (−1.21; 1.07)	0.905	0.55 (−0.17; 1.27)	0.137	0.72 (−0.44; 1.88)	0.222
AGA INFANTS	***IGF2DMR*** ** methylation (n = 434)**				***H19*** ** methylation (n = 445)**			
Birth weight SDS	−0.16 (−0.46; 0.15)	0.310	−0.27 (−0.60; 0.06)	0.111	−0.07 (−0.30; 0.16)	0.533	−0.00 (−0.27; 0.26)	0.982
Δ weight: 2nd trimester – birth	−0.22 (−0.83; 0.39)	0.475	−0.25 (−0.88; 0.37)	0.428	**−0.51 (−0.94; −0.08)**	**0.020**	−0.39 (−0.81; 0.04)	0.076
Δ weight: birth-3 months	−0.31 (−0.73; 0.11)	0.147	−0.26 (−0.69; 0.18)	0.255	**0.32 (0.01; 0.64)**	**0.042**	**0.35 (0.03; 0.68)**	**0.034**
Δ weight: birth-6 months	0.03 (−0.34; 0.40)	0.868	0.05 (−0.36; 0.46)	0.826	0.20 (−0.07; 0.46)	0.143	0.13(−0.18; 0.43**)**	0.414

Results from linear mixed model analyses with DNA methylation as dependent variable and the fetal growth parameters as independent variables. Results are presented for the whole study population and stratified for SGA/AGA. Analyses were performed with square-root transformed methylation data and values are presented as regression coefficients (95% confidence interval).

1 Crude values are adjusted for the correlations between CpG sites, bisulphite batch, and gestational age.

2 Adjusted values were additionally adjusted for maternal characteristics (age, educational level, parity, BMI, folic acid supplement use, smoking and the occurrence of preeclampsia) and fetal gender.

Table 4 shows the associations between genetic variants in the *IGF2/H19* locus and DNA methylation. Genetic variants in the IGF2/H19 locus were associated with both higher (rs3741205, rs2251375) and lower (rs2067051) DNA methylation of *IGF2DMR* (rs3741205, C-allele: β = 1.20, 95%0.71; 1.69, P-value = 2.0×10^−6^, rs2067051, C-allele: β = −0.44, 95% CI −0.86; −0.02, P-value = 0.041 and rs2251375, A-allele: β = 0.48, 95% CI 0.04; 0.93, P-value = 0.033), which corresponds to a standardized difference in DNA methylation of 0.15 SDS (rs3741205), 0.06 SDS (rs2067051) and 0.06 SDS (rs2251375). The genetic variants was not associated with *H19* methylation. The IGF2 SNP (rs3741205) showed a significant association with birth weight (beta 127 grams, 95% CI 40; 215, p-value = 0.004), but the H19 SNPs did not (rs2067051: beta -35 grams,95% CI −109; 39, p-value = 0.349; rs2251375: beta 18 grams,95% CI −60; 97, p-value = 0.643; rs4929984: beta -21 grams,95% CI -96; 53, p-value = 0.572). Next, we explored the possibility of mediation of DNA methylation in the association between the genetic variant rs3741205 and SGA, which is depicted in table 5. The genetic variant rs3741205 was chosen, because of its strong association with *IGF2DMR* DNA methylation. The risk allele of the genetic variant rs3741205 was significantly associated with an increased risk of SGA (adjusted odds ratio (aOR) 1.41, 95% CI 1.24; 1.61, P-value = 1.9×10^−7^). Afterwards, the mean DNA methylation of *IGF2DMR* was added to the model and the effect was attenuated (aOR 1.26, 95% CI 1.10; 1.44, P-value = 0.001).

**Table 4:** Associations between SNPs in the *IGF2/H19* locus and DNA methylation.

		*IGF2DMR* methylation (n = 499)			H19 methylation (n = 510)		
Genetic variant	Gene	bèta^1^	95% CI	P-value	bèta^1^	95% CI	P-value
rs3741205	*IGF2*	**1.20**	**0,71; 1.69**	**2.0 E-6**	−0.06	−0.42; 0.30	0.742
rs2067051	*H19*	**−0.44**	**−0.86; -0.02**	**0.041**	0.28	−0.02; 0.59	0.066
rs2251375	*H19*	**0.48**	**0.04; 0.93**	**0.033**	0.06	−0.26; 0.38	0.717
rs4929984	*H19*	−0.33	−0.75; 0.09	0.123	0.27	−0.04; 0.57	0.084
rs1801131	*MTHFR*	−0.03	−0.45; 0.40	0.898	−0.07	−0.38; 0.24	0.654
rs1801133	*MTHFR*	0.07	−0.35; 0.49	0.752	−0.05	−0.35; 0.25	0.750

Result from linear mixed model analyses with DNA methylation as dependent variable and the genetic variant as independent variable.

1 Analyses were performed with square-root transformed methylation data and adjusted for CpG, bisulphite plate, gestational age at birth and gender.

**Table 5:** Mediating effects of DNA methylation.

	Small for gestational age		
	aOR	95% CI	P-value
Model 1: adjusted for gender			
rs3741205	**1.41**	**1.24; 1.61**	**1.9×10^−7^**
Model 2: model 1 + mean DNA methylation of *IGF2DMR*			
rs3741205	**1.26**	**1.10; 1.44**	**0.001**

Result from logistic regression analyses with small-for-gestational age as dependent variable and the genetic variant as independent variable. aOR: adjusted odds ratio, 95 CI and their corresponding P-value represent the effect of the minor allele (C-allele) on the risk of having a small-for-gestational age infant.

Last, the analyses were repeated with the exclusion of the ADHD cases and subsequently with the exclusion of LGA cases. The results of the ADHD cases are depicted in Table S4. After exclusion of the ADHD cases, the previous found associations remained and the effect estimates did not change substantially. The results of the LGA cases can be found in Table S5. After exclusion of the LGA cases, the previous observed associations remained and the effect estimates did not change substantially.

## Discussion

In 540 children, derived from a population-based birth cohort, we examined whether DNA methylation levels of the *IGF2DMR*, *H19* and *MTHFR* gene in cord blood were associated with fetal and infant growth.

The *IGF2* and *H19* loci have been studied extensively in the past and are strong candidate genes for influencing birth weight [23]. In our study, lower DNA methylation of *IGF2DMR* was observed in children born SGA. There have been conflicting results regarding the association between *IGF2/H19* DNA methylation and fetal and infant growth [28], [29], [30], [31]. A recent study by St-Pierre et al. [31] studied *IGF2/H19* DNA methylation at both the maternal and the fetal side of the placenta in relation to fetal birth anthropometrics. In this study, higher *IGF2DMR* DNA methylation was associated with higher birth weight. Moreover, they showed that alterations in *IGF2/H19* DNA methylation are likely to be functional, because of the established positive association with circulating IGF2 concentrations in cord blood. In addition, Displas et al. reported loss of imprinting of *IGF2/H19* in placentas of children with intrauterine growth restriction [30]. In contrast to our findings, Tobi et al. has found no association between *IGF2DMR* methylation and SGA (difference SGA and AGA −0.2%)[29], which might be explained by their study population of preterm born children (<32 weeks). and the use of a different definition for SGA (<−1 SDS) than ours (<−2SDS). In contrast to previous studies, we included postnatal growth parameters in addition to birth outcomes and found an association between an increase in weight as marker of postnatal growth in the first three months after birth and *IGF2DMR* methylation. As it has been shown that children born AGA and SGA have different postnatal growth patterns, we have shown the postnatal analyses separately. Postnatal growth acceleration has been previously identified as an important risk factor for the development of diseases in later life [32], which could partly be explained by epigenetic reprogramming. In our study, the SGA group is nearly 7 fold smaller than the AGA group. Therefore, a power problem seems a likely explanation for not finding this association in the SGA group. It would be interesting to address this question in future studies.

IGF2 methylation levels determined in umbilical cord white blood cells were positively associated with birth weight [31], [33]. Therefore, the association of SGA lower DNA methylation levels of *IGF2DMR* seems to be in contrast to the concept that increased methylation is associated with transcriptional silencing of the associated gene [5], but is in line with the increase in weight in the first three months after birth and lower *IGF2DMR* methylation levels. Adverse exposures have been previously linked to a decrease in methylation whereas advantageous exposures have been associated with increased methylation [12], [13]. Therefore, DNA methylation can be regarded as a memory of previous exposures and alterations could have consequences in subsequent growth and development. St-Pierre et al [31] showed that 31% of the variance of birth weight is explained by the *IGF2/H19* epigenotype and a genetic variant (rs2107425), which is in linkage disequilibrium (LD) with one of the SNPs that we have investigated, namely rs2251375 (R-squared 1.000).

*IGF2* and *H19* are both imprinted genes and loss of imprinting of the *IGF2/H19* locus has been observed in the Beckwith-Wiedemann syndrome, a congenital overgrowth disorder [34]. In this study, a significant positive association was observed between the polymorphism rs3741205 and *IGF2DMR* methylation. This genetic variant is a defining SNP of a CAGA haplotype in the *IGF2* DMR0 region which has been previously described in patients with a sporadic form of Beckwith-Wiedemann syndrome [35] and the presence of the C-allele has also been positively associated with birth weight [23]. Recently, lower *IGF2 DMR* methylation has been associated with the minor allele of the IGF2 SNP rs2239681, which is in LD with one of the SNPs that we have investigated, namely rs3741205 (R-squared 0.912) [20]. In addition, our results suggest the possibility of mediation of DNA methylation in the association between the genetic variants and SGA. These observations could provide evidence for the complex interplay between the genome, epigenome and environmental factors in growth disorders.

In this study, we found the *MTHFR* region in both groups to be hypomethylated with limited variability. Therefore, no analyses were performed within this region. With the current methods for DNA methylation measurement, we do not recommend for others to investigate this region z

### Methodological considerations

Some strengths and limitations of this study have to be addressed. This study was embedded in a large cohort from whom a selection of Dutch children was studied. As the SGA cases were oversampled in the data, the analyses were performed in all infants and SGA and AGA. The study population also consisted of 92 children with ADHD and 11 children born LGA, which could potentially influence our results. Therefore, all analyses were repeated without the ADHD and LGA children. As this did not change our results substantially, these children were not excluded.

This study showed modest changes in DNA methylation, which remained after multiple testing correction. Our findings are in line with the influences of periconceptional folic acid supplement use and adverse intrauterine exposures, such as the Dutch famine during 1944-45, showing modest epigenetic changes in early and adult life [12], [13]. A recent study by Talens et al [36] has demonstrated that epigenetic changes accumulate over time, both at imprinted (including *IGF2DMR*) and non-imprinted loci. Unfortunately, we were not able to assess whether the DNA methylation variations also result in changes in expression and long-term functional effects. In addition, the issue remains whether the measured DNA methylation differences reflect true methylation changes of the candidate genes of interest. The aim of this study was to estimate differences in the quantitative DNA methylation at selected individual CpG sites of candidate loci. This seems particularly relevant for the *H19* locus, as this is measured around 30%, whereas 50% could be expected. Therefore, it would be valuable to replicate our findings and to validate the chosen methodology. However, others have investigated this region and found comparable levels [12], [20], [31]. Therefore, we believe that our absolute DNA methylation levels are reliable, but most importantly the estimated differences seem to be valid.

DNA methylation was measured in umbilical cord white blood cells and not in other tissues. Therefore, it could be argued that DNA methylation patterns differ in various cell populations [37], [38]. There is a possibility that the differences in DNA methylation could be attributed to the cellular heterogeneity in leukocytes. Unfortunately, no cell count was available for our study population. Therefore, we cannot assess to what extent this has influenced our results. It would be interesting to address this question in future studies. DNA methylation patterns of *IGF2DMR* have been compared in blood and in buccal cells and showed a reasonable correlation (Spearman ρ∼0.5) [37]. It has also been reported that *IGF2DMR* methylation in blood may be informative as it marked the methylation patterns in colon tissue [39]. However, DNA methylation can still differ between tissues. Therefore, it is important to establish in future studies correlations between DNA methylation in peripheral tissues, such as blood, and tissues that are directly involved in the disease. It would be informative to address this question with DNA methylation measurement at a genome-wide level.

### Conclusions

Our analyses suggest that fetal and infant growth are associated with DNA methylation of *IGF2DMR* and *H19.* The observations in this paper could offer support for a potential functional link between DNA methylation in cord blood in the investigated genes and birth outcomes. The understanding how epigenetic control depends on early exposure may shed light on the link between fetal development and health over the lifespan and ultimately suggest new ways to prevent human disease.

## Supporting Information

Table S1 **Details of measured amplicons and PCR primers.** ^1^ Genome built: GRch 37.67. ^2^ Forward and reverse primer that will amplify the bisulphite converted genomic DNA. According to the EpiTyper technology, taqs were added to the 5′end of the primers. Forward primer: 10mer spacer tag is added at the 5′ primer end with the following sequence: 5′-AGGAAGAGAG + primer. Reverse primer: T7 promoter is added to the 5′ primer end with the following sequence: 5′-CAGTAATACGACTCACTATAGGGAGAAGGCT + primer. ^3^ Sequenom, Inc, San Diego, USA
(DOC)

Table S2 **Details quality control.**
(DOC)

Table S3 **Details of genetic variants.** MAF: minor allele frequency, HWE: Hardy-Weinberg equilibrium, * effect allele
(DOC)

Table S4 **DNA methylation and newborn growth parameters, with exclusion of ADHD cases.** Results from linear mixed model analyses with DNA methylation as dependent variable and the fetal growth parameters as independent variables. ADHD cases were excluded for these analyses. Analyses were performed with square-root transformed methylation data and values are presented as regression coefficients (95% confidence interval).
(DOC)

Table S5 **DNA methylation and newborn growth parameters, with exclusion of LGA cases.** Results from linear mixed model analyses with DNA methylation as dependent variable and the fetal growth parameters as independent variables. LGA cases (> 2SDS) were excluded for these analyses. Analyses were performed with square-root transformed methylation data and values are presented as regression coefficients (95% confidence interval).
(DOC)
