# Supplementary material for: DNA Methylation of IGF2DMR and H19 Is Associated with Fetal and Infant Growth: The Generation R Study
Source: PLoS One. 2013 Dec 12;8(12):e81731. doi: 10.1371/journal.pone.0081731 (PMC3861253; doi:10.1371/journal.pone.0081731)
Supplement: Table S1 — Details of measured amplicons and PCR primers. 1 Genome built: GRch 37.67. 2 Forward and reverse primer that will amplify the bisulphite converted genomic DNA. According to the EpiTyper technology, taqs were added to the 5′end of the primers. Forward primer: 10mer spacer tag is added at the 5′ primer end with the following sequence: 5′-AGGAAGAGAG + primer. Reverse primer: T7 promoter is added to the 5′ primer end with the following sequence: 5′-CAGTAATACGACTCACTATAGGGAGAAGGCT + primer. 3 Sequenom, Inc, San Diego, USA (DOC) [file pone.0081731.s001.doc]

**Supplement table S1: Details of measured amplicons and PCR primers**

| **Gene** | **Genomic location^1^** | **Number of CpG units assessed** | **Primer sequence^2^** | **Source** |
| --- | --- | --- | --- | --- |
| Insulin-like growth factor 2 (IGF2) | Chr 11: 2169458-2169796 | 3 CpG units (4 CpG sites) | F: TGGATAGGAGATTGAGGAGAAA | Heijmans 2007 |
|  |  |  | R: AAACCCCAACAAAAACCACT |  |
| H19 | Chr 11: 2019371-2019784 | 10 CpG units (13 CpG sites) | F: GGGTTTGGGAGAGTTTGTGAGGT | Heijmans, 2007 |
|  |  |  | R: ATACCTACTACTCCCTACCTACCAAC |  |
| Methylenetetrahydrofolate reductase (MTHFR) | Chr 1: 11866184-11866627 | 12 CpG units (14 CpG sites) | F: GTTTGTAGTTATTTTTGGTTTTAGTTTT | Designed using the Epidesigner tool^3^ |
|  |  |  | R: TAACCTAAATTCTCCCTCAAATTCC |  |

^1^ Genome built: GRch 37.67

^2^ Forward and reverse primer that will amplify the bisulphite converted genomic DNA. According to the EpiTyper technology, taqs were added to the 5'end of the primers. Forward primer: 10mer spacer tag is added at the 5’ primer end with the following sequence: 5’-AGGAAGAGAG + primer. Reverse primer: T7 promoter is added to the 5’ primer end with the following sequence: 5’-CAGTAATACGACTCACTATAGGGAGAAGGCT + primer

^3^ Sequenom, Inc, San Diego, USA
