# Supplementary material for: DNA Methylation of IGF2DMR and H19 Is Associated with Fetal and Infant Growth: The Generation R Study
Source: PLoS One. 2013 Dec 12;8(12):e81731. doi: 10.1371/journal.pone.0081731 (PMC3861253; doi:10.1371/journal.pone.0081731)
Supplement: Table S2 — Details quality control. (DOC) [file pone.0081731.s002.doc]

**Supplement table S2: details quality control**

| Locus/unit | | Reason for exclusion | Success rate (if included) |
| --- | --- | --- | --- |
| IGF2 DMR | |  |  |
|  | IGF2_01 | Excluded due to rs3741208 |  |
|  | IGF2_02 | Excluded due to rs3741209 |  |
|  | IGF2_03 |  | 91.8% |
|  | IGF2_04 |  | 94.1% |
|  | IGF2_05 | Excluded due to rs4930041 |  |
|  | IGF2_06.07 |  | 94.9% |
|  | IGF2_08 | Excluded due to silent signal |  |
| H19 | |  |  |
|  | H19_01 | Excluded due to duplicate H19_16 and silent signals |  |
|  | H19_02 |  | 95.6% |
|  | H19_03-05 | Excluded due to rs117916983, overlap H19_11 and silent signals |  |
|  | H19_06 | Excluded due to silent signals |  |
|  | H19_07 | Excluded due to silent signal |  |
|  | H19_08 | Excluded due to silent signal |  |
|  | H19_09.10 |  | 95.0% |
|  | H19_11 | Excluded due to overlap H19_03-05 and silent signals |  |
|  | H19_12 |  | 95.4% |
|  | H19_13 |  | 95.1% |
|  | H19_14.15 |  | 96.7% |
|  | H19_16 | Excluded due to duplicate H19_1 and silent signals |  |
|  | H19_17 |  | 95.3% |
|  | H19_18.19 |  | 95.6% |
|  | H19_20 |  | 95.6% |
|  | H19_21 | Excluded due to low mass and silent signal |  |
|  | H19_22 | Excluded due to silent signal |  |
|  | H19_23 | Excluded due to low mass |  |
|  | H19_24 | Excluded due to >25% missing |  |
|  | H19_25 |  | 94.7% |
| MTHFR | |  |  |
|  | MTHFR_01 | Excluded due to >25% missing |  |
|  | MTHFR_02 | Excluded due to low mass, duplicate MTHFR_05 and silent signals |  |
|  | MTHFR_03.04 |  | 92.0% |
|  | MTHFR_05 | Excluded due to low mass, duplicate MTHFR_02 and silent signals |  |
|  | MTHFR_06 | Excluded due to duplicate MTHFR_09 and silent signals |  |
|  | MTHFR_07 |  | 94.6% |
|  | MTHFR_08 |  | 94.6% |
|  | MTHFR_09 | Excluded due to duplicate MTHFR_06 and silent signals |  |
|  | MTHFR_10 | Excluded due to silent signal |  |
|  | MTHFR_11 |  | 94.6% |
|  | MTHFR_12.13 |  | 94.8% |
|  | MTHFR_14 |  | 94.6% |
|  | MTHFR_15 |  | 94.8% |
|  | MTHFR_16-18 | Excluded due to high mass |  |
|  | MTHFR_19 |  | 94.6% |
|  | MTHFR_20 |  | 94.8% |
|  | MTHFR_21 | Excluded due to low mass and silent signal |  |
|  | MTHFR_22 |  | 94.8% |
|  | MTHFR_23 |  | 94.1% |
|  | MTHFR_24 |  | 94.8% |
