# Supplementary material for: DNA Methylation of IGF2DMR and H19 Is Associated with Fetal and Infant Growth: The Generation R Study
Source: PLoS One. 2013 Dec 12;8(12):e81731. doi: 10.1371/journal.pone.0081731 (PMC3861253; doi:10.1371/journal.pone.0081731)
Supplement: Table S3 — Details of genetic variants. MAF: minor allele frequency, HWE: Hardy-Weinberg equilibrium, * effect allele (DOC) [file pone.0081731.s003.doc]

**Supplement table S3: Details of genetic variants**

| rs number | Gene | Minor allele | Major allele | MAF | HWE p-value |
| --- | --- | --- | --- | --- | --- |
| rs3741205 | *IGF2* | C* | A | 0.25 | 0.706 |
| rs2067051 | *H19* | T | C* | 0.48 | 0.451 |
| rs2251375 | *H19* | A* | C | 0.33 | 0.573 |
| rs4929984 | *H19* | A* | C | 0.50 | 0.477 |
| rs1801131 | *MTHFR* | T* | C | 0.30 | 0.482 |
| rs1801133 | *MTHFR* | A* | C | 0.31 | 0.472 |

MAF: minor allele frequency, HWE: Hardy-Weinberg equilibrium, * effect allele
