# Supplementary material for: DNA Methylation of IGF2DMR and H19 Is Associated with Fetal and Infant Growth: The Generation R Study
Source: PLoS One. 2013 Dec 12;8(12):e81731. doi: 10.1371/journal.pone.0081731 (PMC3861253; doi:10.1371/journal.pone.0081731)
Supplement: Table S5 — DNA methylation and newborn growth parameters, with exclusion of LGA cases. Results from linear mixed model analyses with DNA methylation as dependent variable and the fetal growth parameters as independent variables. LGA cases (> 2SDS) were excluded for these analyses. Analyses were performed with square-root transformed methylation data and values are presented as regression coefficients (95% confidence interval). (DOC) [file pone.0081731.s005.doc]

**Supplement Table S5: DNA methylation and newborn growth parameters, with exclusion of LGA cases**

|  | **IGF2 DMR (n=491)** | | |  | **H19 (n=502)** | | |
| --- | --- | --- | --- | --- | --- | --- | --- |
|  | bèta | 95% CI | P-value |  | bèta | 95% CI | P-value |
| MODEL: *adjusted for correlations between CpG sites, bisulphite batch, maternal characteristics (age, educational level, parity, BMI, folic acid supplement use, smoking and the occurrence of preeclampsia) and fetal gender* |  |  |  |  |  |  |  |
| Small-for-gestational age (<-2 SDS) | **-1.07** | **-1.93; -0.22** | **0.014** |  | -0.28 | -0.95; 0.39 | 0.411 |
| Birth weight SDS | 0.08 | -0.19; 0.34 | 0.567 |  | 0.07 | -0.15; 0.28 | 0.539 |
| Δ weight 2nd trimester – birth | -0.08 | -0.60; 0.44 | 0.762 |  | -0.18 | -0.54; 0.19 | 0.346 |
| Δ weight birth-3 months | **-0.50** | **-0.90; 0.10** | **0.013** |  | 0.21 | -0.10; 0.51 | 0.179 |
| Δ weight birth-6 months | -0.17 | -0.51; 0.17 | 0.316 |  | 0.10 | -0.15; 0.36 | 0.428 |

Results from linear mixed model analyses with DNA methylation as dependent variable and the fetal growth parameters as independent variables. LGA cases (> 2SDS) were excluded for these analyses. Analyses were performed with square-root transformed methylation data and values are presented as regression coefficients (95% confidence interval).
